# Pre-existing parasympathetic dominance seems to cause persistent heart rate slowing after 6 months of fingolimod treatment in patients with multiple sclerosis

**DOI:** 10.1007/s10286-024-01073-w

**Published:** 2024-10-09

**Authors:** Max J. Hilz, Francesca Canavese, Carmen de Rojas-Leal, De-Hyung Lee, Ralf A. Linker, Ruihao Wang

**Affiliations:** 1 Department of Neurology, University of Erlangen-Nuremberg, Schwabachanlage 6, 91054 Erlangen, Germany; 2 Department of Neurology, Icahn School of Medicine at Mount Sinai, New York, NY USA; 3 Department of Neurology, Hospital Universitario Virgen de La Victoria, Malaga, Spain; 4 Department of Neurology, University Hospital Regensburg, University of Regensburg, Regensburg, Germany

**Keywords:** Fingolimod, Relapsing–remitting multiple sclerosis, Parasympathetic predominance, Heart rate slowing, Sphingosine-1-phosphate receptor, Active standing

## Abstract

**Purpose:**

Vagomimetic fingolimod effects cause heart rate (HR) slowing upon treatment initiation but wear off with sphingosine-1-phosphate receptor downregulation. Yet, prolonged HR slowing may persist after months of fingolimod treatment. We evaluated whether cardiovascular autonomic modulation differs before and 6 months after fingolimod initiation between patients with RRMS with and without initially prolonged HR slowing upon fingolimod initiation.

**Methods:**

In 34 patients with RRMS, we monitored RR intervals (RRI) and blood pressure (BP), at rest and upon standing up before fingolimod initiation. Six hours and 6 months after fingolimod initiation, we repeated recordings at rest. At the three time points, we calculated autonomic parameters, including RRI standard deviation (RRI-SD), RRI-total-powers, RMSSD, RRI high-frequency [HF] powers, RRI and BP low-frequency (LF) powers, and baroreflex sensitivity (BRS). Between and among patients with and without prolonged HR slowing upon fingolimod initiation, we compared all parameters assessed at the three time points (analysis of variance [ANOVA] with post hoc testing; significance: *p* < 0.05).

**Results:**

Six hours after fingolimod initiation, all patients had decreased HRs but increased RRIs, RRI-SDs, RMSSDs, RRI-HF-powers, RRI-total-powers, and BRS; 11 patients had prolonged HR slowing. Before fingolimod initiation, these 11 patients did not decrease parasympathetic RMSSDs and RRI-HF-powers upon standing up. After 6 months, all parameters had reapproached pretreatment values but the 11 patients with prolonged HR slowing had lower HRs while the other 23 patients had lower parasympathetic RMSSDs and RRI-HF-powers, and BRS than before fingolimod initiation.

**Conclusion:**

Our patients with prolonged HR slowing upon fingolimod initiation could not downregulate cardiovagal modulation upon standing up even before fingolimod initiation, and 6 months after fingolimod initiation still had more parasympathetic effect on HR while cardiovagal modulation and BRS were attenuated in the other 23 patients. Pre-existing parasympathetic predominance may cause prolonged HR slowing upon fingolimod initiation.

## Introduction

### Cardiovascular effects of initial and long-term fingolimod application

The sphingosine-1-phosphate (S1P) receptor modulator fingolimod was the first oral disease-modifying drug for the treatment of relapsing–remitting multiple sclerosis (RRMS) [1]. Initially, fingolimod has vagomimetic and also some vasodilating effects that cause a transient decrease in heart rate (HR) and blood pressure (BP) upon fingolimod initiation [2–11]. Usually, fingolimod slows HR by 10–15 beats per minute with a nadir after 4–5 h and attenuation beginning after 6 h [1, 3–9]; HR slowing is commonly resolved after 24 h [1, 10, 12–14]. More severe side effects such as bradycardia occur in 0.5–2.4%, serious cardiovascular adverse events in 0.9%, and atrioventricular (AV) blocks in 0.4% of patients [1, 3].

The cardiovascular effects result from fingolimod’s function as an S1P receptor modulator [5, 12, 15–17]. Both the prodrug fingolimod and active fingolimod phosphate [16] have structural similarity to the endogenous lysophospholipid sphingosine and S1P, respectively [10, 16, 18, 19]. S1P binds to five different S1P receptors, S1PR 1–5 [10, 15, 16, 20–22], each of which is coupled to a G-protein complex and conveys receptor-specific functions which include immune responses, inflammation, angiogenesis, cell differentiation and migration, endothelial function and angiogenesis or modulation of heart, blood vessel tone, and blood pressure [10, 20, 23, 24].

The phosphorylated* S* enantiomer of fingolimod (fingolimod-P) binds to S1P receptors 1, 3, 4, and 5 with its highest binding affinity to S1P1 receptors [15]. Fingolimod-P displaces endogenous S1P from S1P1 receptors [25] and initially exhibits agonistic effects at S1P1 receptors [10, 15, 16]. However, the overstimulation of S1P1 receptors with fingolimod-P induces S1P1 receptor desensitization and endocytosis with degradation of S1P1 receptor–ligand complexes [10, 15, 26–28]. This fingolimod-induced downregulation and reduction of S1P1 receptors on cell surfaces accounts for decreased signal transduction via endogenous S1P. Hence, the initially agonistic fingolimod-P turns into a functional antagonist of S1P as the S1P1 receptor downregulation and reduction inhibits lymphocyte egress from secondary lymphoid organs [10, 15, 16] and their migration into the central nervous system [20, 29]. The initially agonistic fingolimod effects on S1P1 receptors also induce a transient BP decrease due to S1P1 receptor-mediated activation of endothelial nitric oxide (NO) synthetase with subsequent NO release and vasodilation [10, 15, 16]. Clinically more prominent are the aforementioned initial and transient slowing of HR and atrioventricular conduction via fingoliomod’s initially agonistic effect on S1P1 receptors [5, 10, 16, 17, 30, 31]. S1P1 receptors are prominent in cardiomyocytes and endothelial cells of cardiac vessels [15, 32], and they modulate the function of atrial myocytes and thus heart rate [10, 15–17, 27]. Binding of endogenous S1P or of the initially agonistic S1P receptor modulator fingolimod to atrial G-protein-coupled S1P1 receptors catalyzes the opening of G-protein-gated inwardly rectifying potassium channels (GIRK channels); the subsequent potassium efflux from cardiomyocytes hyperpolarizes cell membranes which reduces myocyte excitability [17, 27, 33, 34]. The resulting negative chronotropic and dromotropic effects are similar to those induced by parasympathetically released acetylcholine [10, 15, 16, 27] which activates the same G-protein-gated cascade used by S1P1 receptors but binds to cardiac muscarinic, G-protein-coupled M2 receptors to induce GIRK channel opening and potassium efflux [10, 15, 16].

### Possible autonomic contribution to swift recovery of negative chronotropic and dromotropic fingolimod effects

Negative chronotropic and dromotropic fingolimod effects are usually transient [6, 10, 15–17, 35, 36] and only occur upon fingolimod initiation or upon fingolimod reinitiation after a treatment pause [1, 12, 20, 35], presumably because agonistic fingolimod effects on S1P1 receptors wane while S1P1 receptor desensitization and internalization starts several hours after fingolimod initiation [10, 16, 17, 35, 36].

Usually, cardiovascular effects are asymptomatic and dissolve within 24 h after fingolimod initiation [1, 12, 36]. In their analysis of first-dose fingolimod effects and safety data of three phase 3 studies, DiMarco et al. therefore consider other mechanisms that might add to the compensatory effects of S1P1 receptor internalization and downregulation on the recovery of HR and AV conduction slowing, including influences of the autonomic nervous system on HR and AV conduction [36].

### Is autonomic dysfunction contributing to prolonged negative chronotropic fingolimod effects?

In fact, several studies showed persistent HR slowing in patients with RRMS after several months or even after more than 1 year of fingolimod treatment [37–40], and some authors consider centrally mediated autonomic cardiovascular effects to contribute to persistent HR changes [37–39]. More than 1 year after fingolimod initiation, Akbulak et al. recorded slower HRs than before fingolimod initiation in 64 patients with RRMS and assumed that patients with bradycardia-related adverse events might have enhanced parasympathetic regulation [38]. Six months after fingolimod initiation, Racca et al. found no significant difference in HR of 21 patients with RRMS but a significant decrease in parameters reflecting sympathetic and parasympathetic cardiac modulation compared to pretreatment values [39]. Three months after fingolimod initiation, Simula et al. found no difference in daytime HRs compared to corresponding pre-treatment values in their 24 patients with RRMS while nighttime HRs were still lower than pretreatment nighttime HRs [37]. The authors assumed that nocturnal parasympathetic predominance facilitated additional vagomimetic fingolimod effects and thus the additional nighttime slowing of HR [37]. Rossi et al. assessed autonomic cardiac modulation in 55 patients with RRMS at rest, during head-up tilt, Valsalva maneuver (VM), deep breathing, and sustained handgrip exercise before fingolimod initiation and found correlations between several pre-treatment autonomic parameters and values of HR, electrocardiographic PR and QT intervals assessed during the first 6 h after fingolimod initiation [41]. Fingolimod-induced bradycardia correlated significantly with the parasympathetic indices Valsalva ratio and the expiratory–inspiratory HR difference during deep breathing while sympathetic parameters correlated negatively with the increase in PR intervals [41]. Again, the authors concluded that sinus bradycardia upon fingolimod initiation is associated with increased parasympathetic function while bradyarrhythmia is associated with reduced sympathetic function [41].

Finally, our group concluded from a study of 21 patients with RRMS that autonomic parameters may serve as predictors of prolonged HR slowing upon fingolimod initiation [42]. Seven of our 21 patients had prolonged HR slowing after fingolimod initiation; in these seven patients, sustained handgrip exercise generated a higher sympathetically mediated BP increase than in the 14 patients with HR reacceleration within the first 6 h. In the seven patients, VM moreover unveiled exaggerated parasympathetic HR slowing upon strain release. We concluded that MS-related, subclinical central autonomic dysfunction contributes to delayed HR reacceleration upon fingolimod initiation [42].

### So far, no study tested whether pre-existing central autonomic dysfunction might account for long-lasting HR slowing or enduring autonomic changes during fingolimod treatment

Yet, cardiovascular dysregulation due to MS-related impairment of the central autonomic network (CAN) [42, 43] might not only account for delayed HR reacceleration after the first dose of fingolimod but lesion-related dysfunction is likely to persist [44] and might therefore contribute to autonomic imbalance and HR slowing even months or years after fingolimod initiation as previously suggested [37, 38]. Therefore, we tested the hypothesis that autonomic dysfunction existing in patients with RRMS prior to fingolimod treatment may contribute to HR slowing and altered cardiovascular autonomic modulation even after several months of fingolimod treatment. Hence, we assessed cardiovascular and autonomic parameters in patients with RRMS before and 6 h after fingolimod initiation, and identified patients with prolonged HR slowing after the first dose of fingolimod in order to determine whether HR, BP, and autonomic parameters differed between patients with and without prolonged HR slowing not only after the first 6 h but also after 6 months of fingolimod treatment, and whether such differences might be associated with pre-existing differences in central autonomic modulation between both patient groups [42].

## Patients and methods

We enrolled patients with relapsing–remitting MS (RRMS) who were about to start a disease-modifying therapy (DMT) with 0.5 mg fingolimod per day [42]. The fingolimod treatment followed the recommendations of the European Medicines Agency and included continuous HR monitoring for at least 6 h after fingolimod initiation or longer if HR had reached its lowest value 6 h after the first dose (http://www.ema.europa.eu/ema) [42].

To determine possible long-term effects of fingolimod on HR and on cardiovascular autonomic modulation, we intended to identify patients with prolonged HR reacceleration upon fingolimod initiation, i.e., patients in whom the lowest HR value was recorded 6 h after fingolimod initiation and who therefore required further HR monitoring [42]. Then, HR, systolic and diastolic blood pressure (BPsys, BPdia), respiration and autonomic parameters were reassessed in all patients during a follow-up examination after 6 months of daily fingolimod treatment.

To determine whether there were pre-existing subclinical autonomic changes or differences between patients with and without prolonged HR slowing even before fingolimod initiation, we evaluated autonomic cardiovascular modulation before fingolimod initiation under resting condition and during active standing up. Six hours and 6 months after fingolimod initiation, we only calculated autonomic cardiovascular parameters under resting conditions in order not to compromise patient cooperation.

To elucidate possible persistence and mechanisms of prolonged HR slowing due to fingolimod, values of all parameters (see below) assessed in patients with prolonged HR slowing and patients without prolonged HR slowing were compared between both patient groups and within each group before fingolimod initiation, 6 h, and 6 months after fingolimod initiation.

The Ethics Committee of the University of Erlangen-Nuremberg had approved the study proposal, and the study was registered at the German Clinical Trial Register (DRKS00004548) [42]. Before participating in the study, all participants gave written informed consent after detailed explanation of the study protocol, its purpose, all procedures, and any potential risks of all procedures used, according to the Declaration of Helsinki [42].

Patients who had received previous DMT were taken off their previous medication for at least the period consistent with current recommendation [45], i.e., for at least 6 months if the patients were on cytotoxic drugs (e.g., mitoxantrone) and for at least 2–3 months if the patients were on natalizumab [42]. Fingolimod could generally be started immediately after discontinuation of interferon or glatiramer acetate [42].

To ensure that we only tested patients in whom any finding of autonomic dysfunction could not be ascribed to other causes than the hypothesized MS-related dysfunction of the CAN [42], we excluded participants with other diseases than MS or risk factors affecting the autonomic nervous system, with clinically overt signs of autonomic dysfunction, or on medication possibly affecting autonomic function [42]. Furthermore, we excluded patients with MS and high degrees of disability, such as spasticity, severe motor impairment, or prominent sensory loss [42]. Moreover, we only enrolled participants who had a normal electrocardiogram (ECG).

In analogy of our previously reported studies [11, 42], we tested all participants between 9 AM and 2 PM, after a resting period of at least 40 min, which ensured a stable cardiovascular situation [42]. All bio-signal recordings were performed under standardized conditions in a quiet room with an ambient temperature of 24 °C and stable humidity [42].

All patients received their first fingolimod dose immediately after the first recording of HR, BP, and respiration at supine rest. As previously described [11, 42], we estimated MS severity using the expanded disability status scale (EDSS) [46] and the multiple sclerosis functional composite (MSFC) [47], a scoring system that consist of a timed 25-foot walk, the 9-hole peg test, and a paced auditory serial addition test [48, 49].

### Bio-signal recordings prior to and 6 h as well as 6 months after fingolimod initiation

In the patients with RRMS, we performed bio-signal recordings at supine rest before fingolimod initiation, 6 h, and 6 months after fingolimod initiation. We monitored HR [bpm] and electrocardiographic RR intervals (RRI; [ms]) using 3-lead electrocardiography, beat-to-beat BPsys and BPdia [mmHg] using finger pulse photoplethysmography (Portapress; TPD Biomedical Instrumentation, Amsterdam, the Netherlands), and respiratory frequency (RESP [min^−1^]) using chest impedance measurements [50, 51]. We also determined the difference between baseline HR before fingolimod initiation and the lowest HR during the first 6 h after fingolimod initiation.

For signal recordings, we used a custom-designed data acquisition and analysis system (SUEmpathy™, SUESS-Medizintechnik, Germany) to sample and digitize bio-signals and to display signal recordings on a personal computer [42]. Data were stored for off-line analysis [50]. For further analyses, we selected 3-min signal recordings that were free of artifacts from which we extracted the most stationary 120-s epochs to calculate the averaged value for each signal and each derived autonomic parameter [42].

### Calculation of time- and frequency-domain parameters of cardiovascular autonomic modulation

From the 120-s bio-signal epochs, we calculated two time-domain parameters reflecting sympathetic and parasympathetic cardiac modulation, the standard deviation of RRIs (RRI-SD) and the coefficient of variation of RRIs (RRI-CV) [11, 42, 50–52]. We also calculated the square root of the mean squared differences of successive RRIs (RMSSD) as index of parasympathetic cardiac modulation [11, 42, 50–52].

In addition, we determined frequency-domain parameters of sympathetic and parasympathetic modulation of RRI and BP [11, 42]. We used trigonometric regressive spectral analyses (TRS) to assess slow, underlying RRI and BP oscillations in the so-called low-frequency (LF; 0.04–0.14 Hz) and high-frequency (HF; 0.15–0.50 Hz) ranges that represent autonomic RRI and BP modulation [50, 52, 53].

LF oscillations of RRI at rest reflect sympathetic modulation and, to an undetermined degree, also parasympathetic modulation of HR or RRIs [50, 52, 53]. In contrast, LF oscillations of BP are related to sympathetic outflow only [50–52]. HF oscillations of RRIs only reflect parasympathetic modulation [50–52], while BP fluctuations in the HF range are primarily a mechanical consequence of respiration-induced fluctuations in venous return and cardiac output [50–52]. We determined the magnitude of LF and HF oscillations as the integral under the power spectral density curves of RRI (ms^2^/Hz) and BP (mmHg^2^/Hz) for the LF and HF frequency bands, and expressed the magnitude as LF and HF powers of RRI (ms^2^) and BP (mmHg^2^) [11, 42, 50, 53].

We further used the sum of LF and HF powers as an approximate measure of the total power (TP) of RRI oscillations, i.e., as index of the overall cardiac autonomic modulation [11, 42, 50–52]. Moreover, we calculated the ratio between RRI oscillations in the LF and HF ranges, and used the RRI-LF/HF ratio as parameter reflecting the balance between sympathetic and parasympathetic influences on HR modulation [52, 54].

Finally, we assessed baroreflex sensitivity (BRS) using the TRS software which selects pairs of LF and HF oscillations of BPsys and RRI with high coherence [55]. Coherence between two signals oscillating at a specific frequency may span from 0, i.e., no association, to 1, i.e., maximum association [56]. High coherence of two signals at the specific frequency, e.g., above 0.7, indicates a stable phase relation—and thus synchronization—between the oscillations of the two signals at this specific frequency [56]. From the selected pairs of coherent LF and HF oscillations of BPsys and RRI, the TRS software derives the sensitivity of the baroreflex loop (ms·mmHg^−1^) as gain values from changes in RRIs (ms) in relation to changes in BPsys (mmHg) [11, 42, 50, 57].

It must be noted that the above parameters are markers of cardiovascular autonomic modulation provided there are no interferences such as drugs that reduce or increase the sympathetic or parasympathetic modulation, or—as in this study—drugs that have an initially vagomimetic effect. Then, the above parameters reflect a combined effect of autonomic and pharmacologic influences on cardiovascular modulation.

### Additional assessment of autonomic responses to sympathetic and parasympathetic challenge maneuvers prior to fingolimod initiation

Prior to fingolimod initiation, we determined whether there were any patients with pre-existing cardiovascular autonomic dysregulation upon orthostasis since such dysfunction might be associated with a delay in HR reacceleration upon fingolimod initiation [42]. After 10 min supine rest, patients stood up [42, 51], and we assessed supine and standing values of HR, BPsys, BPdia, respiration and sympathetic activation and parasympathetic withdrawal upon baroreflex unloading using the spectral powers of RRI and BP modulation mentioned above [54, 55].

Metronomic deep breathing (MDB) was performed to assess parasympathetic activation upon breathing deeply for 3 min at six cycles per minute [42, 51]. To assess the maximal HR increase during inspiration and the maximal HR decrease during expiration [42, 51], we calculated the ratio between the longest RRI during expiration and the shortest RRI during inspiration, i.e., the RRI-E/I ratio [42, 51].

The Valsalva maneuver (VM) was used to determine the cardiovagal buffer capacity in response to baroreflex loading in response to the BP overshoot upon strain release. During VM, participants maintained an expiratory pressure of 40 mmHg for 15 s [42, 51]. We calculated the Valsalva ratio (VR) as the ratio between the highest and lowest RRIs during the first 30 s after strain release as index of the baroreflex-mediated parasympathetic activation [42, 51, 58].

### Statistical analysis

We used the Shapiro–Wilk test to test data for normal distribution. We assessed differences between bio-signals and autonomic parameters determined before fingolimod initiation, 6 h after fingolimod initiation, and after 6 months of daily fingolimod treatment within and between the patient groups with and without prolonged HR slowing upon fingolimod initiation by means of analysis of variance (ANOVA) for repeated measurements (general linear model). We defined “recordings” (before, 6 h, and 6 months after fingolimod initiation) as within-subject factor, and “groups” (patients with and patients without prolonged HR slowing upon fingolimod initiation) as between-subject factor. Mauchly’s test of sphericity assessed the suitability of the ANOVA. We employed the Greenhouse–Geisser correction in case of violation of the sphericity assumption. For each subgroup, we determined differences between pairs of values assessed at two of the three time points, i.e., values prior to fingolimod initiation, and values assessed 6 h and 6 months after fingolimod initiation, and used post hoc paired Student’s* t* tests for normally distributed data or Wilcoxon tests for non-normally distributed data.

Between the patients with and those without delayed HR reacceleration, we compared values at each of the three time points using two-sided* t* tests for independent samples for normally distributed data or Mann–Whitney* U* tests for non-normally distributed data. Similarly, we compared differences between baseline HRs before fingolimod initiation and HRs during the first 6 h after fingolimod initiation between patients with and patients without prolonged HR slowing using* t* test for independent samples in case of normally distributed data or Mann–Whitney* U* tests in case of non-normally distributed data.

We used the same tests to compare the autonomic indices assessed prior to fingolimod initiation during active standing between patients with and patients without prolonged HR slowing.

We applied the Fisher’s exact test to assess gender differences between groups. Significance was assumed for* p* values below 0.05. For data analysis, we used a commercially available statistical program (IBM SPSS Statistics for Windows, Version 26. Armonk, NY, USA).

## Results

### Demographic and clinical data of the patients with RRMS

Thirty-four patients (18 women; mean age 33.7 years, standard deviation [SD] 10.2 years) with RRMS participated in all recordings. In 23 patients with RRMS, HR slowing upon fingolimod initiation reaccelerated within 6 h upon fingolimod intake. Eleven patients with RRMS had prolonged HR slowing after fingolimod initiation. Gender distribution, age, and EDSS and MSFC values reflecting clinical MS severity did not differ significantly between the 11 and the 23 patients (Table 1).

**Table 1:** Demographic and clinical data of 23 patients without prolonged heart rate slowing and 11 patients with prolonged heart rate slowing after fingolimod initiation

	23 patients without prolonged heart rate slowing	11 patients with prolonged heart rate slowing	*p* values
Gender (men/women)	9/14	7/4	0.274
Age [year]	34.9 ± 10.3	31.1 ± 10.0	0.324
Disease duration [year]	3.8 (2.0–8.3)	2.6 (1.7–6.6)	0.424
EDSS before fingolimod initiation	2.41 ± 1.20	1.73 ± 0.56	0.114
EDSS after 6 months of fingolimod therapy	2.19 ± 1.33	2.14 ± 0.78	0.506
Comparison between first and second EDSS values	*p* = 0.085	*p* = 0.066	
MSFC before fingolmod initiation	0.11 ± 0.41	0.13 ± 0.35	0.902
MSFC after 6 months of fingolimod therapy	0.31 ± 0.44	0.37 ± 0.35	0.718
Comparison between first and second MSFC values	*p* = 0.001*	*p* = 0.001*	

Values of EDSS and MSFC are expressed as mean ± standard deviation. Disease duration is expressed as median (interquartile range)

*EDSS* expanded disability status scale, *MSFC* multiple sclerosis functional composite

*Statistically significant (as *p* < 0.05)

After 6 months of fingolimod treatment, EDSS scores and MSFC values still were similar between both patient groups. Moreover, EDSS scores of each subgroup did not differ from the group’s EDSS scores prior to fingolimod initiation (Table 1). In contrast, 6 months MSFC values were significantly higher within both groups than their MSFC values prior to fingolimod initiation (*p* = 0.001; Table 1).

### Bio-signals and autonomic parameters before fingolimod initiation

Before fingolimod initiation, values of HR, BPsys, BPdia, respiratory frequency, and autonomic parameters assessed at supine rest did not differ between patients with and those without prolonged HR slowing (Tables 2 and 3). However, upon standing up, only the 23 patients without prolonged HR slowing but not the 11 patients with prolonged HR slowing had a slight, significant BPsys and BPdia increase (Table 3). Moreover, parasympathetically mediated RMSSD and RRI-HF-powers decreased significantly only in the 23 patients (*p* < 0.001) but not the 11 patients upon standing (Tables 2 and 3). Furthermore, the index of sympathetic–parasympathetic balance, the RRI-LF/HF ratios increased significantly in the 23 patients (*p* < 0.001) but not in the 11 patients with prolonged HR slowing (Table 3). Values of all other parameters showed similar changes from supine to standing or remained stable in both groups (Tables 2 and 3). During MDB, RRI-E/I ratios did not differ between the 11 patients with MS (1.4 ± 0.1) and the 23 patients (1.5 ± 0.2; *p* = 0.133). Moreover, Valsalva ratios were similar in the 11 patients (1.8 ± 0.4) and the 23 patients (1.8 ± 0.5; *p* = 0.882).

**Table 2:** Comparison of bio-signals, time-domain parameters at rest and upon active standing in 23 patients without prolonged heart rate slowing and 11 patients with prolonged heart rate slowing

		23 patients without prolonged heart rate slowing	11 patients with prolonged heart rate slowing	Comparison between two groups
Respiration [cpm]	Sitting	13.8 ± 3.6	14.2 ± 3.4	*p* = 1.000
	Standing	13.5 ± 4.4	14.3 ± 3.2	*p* = 0.558
	*p* value	0.683	0.804	
RRI [ms]	Sitting	780.0 ± 80.0	769.7 ± 78.4	*p* = 0.402
	Standing	670.2 ± 72.0	673.0 ± 63.8	*p* = 0.918
	*p* value	< 0.001*	0.002*	
BPsys [mmHg]	Sitting	117.5 ± 13.5	124.6 ± 21.3	*p* = 0.270
	Standing	126.0 ± 12.4	132.3 ± 20.2	*p* = 0.280
	*p* value	0.007*	0.128	
BPdia [mmHg]	Sitting	63.1 ± 11.2	68.7 ± 19.0	*p* = 0.268
	Standing	72.0 ± 9.4	76.8 ± 14.3	*p* = 0.268
	*p* value	0.008*	0.057	
RRI-SD [ms]	Sitting	39.9 ± 16.7	38.7 ± 12.7	*p* = 0.719
	Standing	36.1 ± 9.6	41.5 ± 22.9	p = 0.338
	*p* value	0.309	0.375	
RRI-CV [%]	Sitting	5.1 ± 2.0	5.0 ± 1.6	*p* = 0.851
	Standing	5.4 ± 1.5	6.1 ± 3.1	*p* = 0.400
	*p* value	0.545	0.124	
RMSSD [ms]	Sitting	30.2 ± 16.4	25.5 ± 11.8	*p* = 0.194
	Standing	16.5 ± 5.4	18.8 ± 10.4	*p* = 0.398
	*p* value	< 0.001*	0.171	

Student’s *t* and paired* t* test for normally distributed data, Mann–Whitney *U* test und Wilcoxon test for non-normally distributed data

*RRI* RR interval, *BPsys* systolic blood pressure, *BPdia* diastolic pressure, *RESP* respiratory frequency, *RRI-SD* standard deviation of RRI, *RRI-CV* coefficient of variation of RRI, *RMSSD* square root of the mean squared differences of successive RRIs

*Statistically significant (as *p* < 0.05)

**Table 3:** Comparison of frequency-domain parameters at rest and upon active standing in 23 patients without prolonged heart rate slowing and 11 patients with prolonged heart rate slowing

		23 patients without prolonged heart rate slowing	11 patients with prolonged heart rate slowing	*p* value
RRI-LF-powers [ms^2^]	Sitting	1147.2 ± 941.5	1076.0 ± 711.7	0.985
	Standing	939.2 ± 444.7	1704.3 ± 1698.0	0.550
	*p* value	0.648	0.059	
RRI-HF-powers [ms^2^]	Sitting	428.5 ± 430.8	326.9 ± 350.6	0.223
	Standing	127.2 ± 115.9	188.5 ± 233.6	0.954
	*p* value	< 0.001*	0.445	
RRI-total-powers [ms^2^]	Sitting	1575.7 ± 1300.4	1402.9 ± 906.0	1.000
	Standing	1066.3 ± 500.2	1892.8 ± 1915.5	0.630
	*p* value	0.301	0.203	
RRI-LF/HF ratio	Sitting	4.8 ± 3.9	9.3 ± 14.1	0.118
	Standing	20.0 ± 34.9	12.9 ± 6.5	0.603
	*p* value	< 0.001*	0.093	
BPsys-LF-powers [mmHg^2^]	Sitting	16.4 ± 8.7	19.9 ± 12.2	0.287
	Standing	33.1 ± 18.8	48.4 ± 39.0	0.499
	*p* value	< 0.001*	0.022*	
BPsys-HF-powers [mmHg^2^]	Sitting	3.5 ± 2.0	4.4 ± 3.1	0.615
	Standing	5.7 ± 3.1	6.9 ± 7.0	0.773
	*p* value	0.002*	0.575	
BRS [ms/mmHg]	Sitting	7.4 ± 2.4	6.9 ± 3.5	0.183
	Standing	4.5 ± 1.6	4.2 ± 1.7	0.576
	*p* value	< 0.001*	0.028*	

Student’s *t* and paired* t* test for normally distributed data, Mann–Whitney *U* test und Wilcoxon test for non-normally distributed data

*RRI* RR interval, *HF* high frequency, *LF* low frequency, *BPsys* systolic blood pressure, *BRS* baroreflex sensitivity

*Statistically significant (as *p* < 0.05)

### Bio-signals and autonomic parameters 6 h after fingolimod initiation

Six hours after fingolimod initiation, both patient groups had significantly lower HRs and higher RRI values than before fingolimod initiation. However, in the 11 patients with prolonged HR slowing, 6-h HRs were significantly lower and RRI values significantly higher than in the 23 patients without prolonged HR slowing (HR 58.2 ± 7.8 bpm vs. 67.2 ± 7.9 bpm; *p* = 0.005; RRIs 1048.0 ± 139.0 ms vs. 905.0 ± 110.1 ms; Table 4). Moreover, the maximum HR decrease, i.e., the difference between pretreatment HR and HR 6 h after fingolimod initiation, was greater in the 11 patients with than in the 23 patients without prolonged HR slowing (22.0 ± 5.5 vs. 15.2 ± 7.1 bpm, *p* = 0.011). After 6 h, BPsys and BPdia values of both patient groups did not differ significantly from their pre-fingolimod values (Table 4). After 6 h, the total powers of RRI modulation were higher in the 11 patients than in the 23 patients (2724.8 ± 1176.4 vs. 2434.4 ± 2569.1 ms^2^; *p* = 0.042; Table 6). All other 6-h parameters were similar between the two patient groups. However, in both groups not only RRIs and RRI-total-powers but also RRI-SD values, parasympathetic RMSSD values, RRI-HF-powers, and BRS values were higher 6 h after than before fingolimod initiation (Tables 4, 5, 6, and 7).

**Table 4:** Bio-signals of 23 patients without prolonged heart rate slowing (PHRS) and 11 patients with PHRS upon fingolimod initiation at three time points: before fingolimod initiation, 6 h after fingolimod initiation, and after 6 months of continuous fingolimod therapy

Parameter	Group	Time points			*p* values			
		Before fingolimod (1)	6 h after fingolimod (2)	6 months after fingolimod (3)	1 vs. 2	2 vs. 3	1 vs. 3	RANOVA
Resp [cmp]	Without PHRS	13.8 ± 3.6	15.3 ± 3.4	12.6 ± 4.3	0.302	0.100	0.102	*p* = 0.089
	With PHRS	14.2 ± 3.4	14.6 ± 4.1	14.1 ± 4.0	0.775	0.756	0.927	
	Post hoc analysis not performed (ANOVA: *p* > 0.05)							
Heart rate [bpm]	Without PHRS	77.8 ± 7.8	67.2 ± 7.9	76.8 ± 10.6	< 0.001*	< 0.001*	0.690	*p* < 0.001*
	With PHRS	78.7 ± 7.9	58.2 ± 7.8	70.4 ± 9.8	< 0.001*	0.007*	0.049*	
	*p* values	0.778	0.005*	0.104				
RRI [ms]	Without PHRS	778.1 ± 77.8	905.0 ± 110.1	795.4 ± 107.3	< 0.001*	< 0.001*	0.533	*p* < 0.001*
	With PHRS	769.7 ± 78.4	1048.0 ± 139.0	865.9 ± 111.5	< 0.001*	0.003*	0.034*	
	*p* values	0.722	0.003*	0.086				
BPsys [mmHg]	Without PHRS	117.5 ± 13.5	114.6 ± 12.2	117.0 ± 19.6	0.467	0.433	0.978	*p* = 0.886
	With PHRS	124.6 ± 21.3	126.7 ± 16.0	123.1 ± 17.0	0.925	0.802	0.822	
	Post hoc analysis not performed (ANOVA: *p* > 0.05)							
BPdia [mmHg]	Without PHRS	63.1 ± 11.2	57.7 ± 7.6	65.8 ± 13.3	0.056	0.002*	0.482	*p* = 0.011*
	With PHRS	68.7 ± 19.0	61.0 ± 11.8	70.1 ± 11.2	0.190	0.024*	0.832	
	*p* values	0.289	0.343	0.362				

*Statistically significant (as *p* < 0.05)

**Table 5:** Time-domain parameters of 23 patients without prolonged heart rate slowing (PHRS) and 11 patients with PHRS upon fingolimod initiation at three time points: before fingolimod, 6 h and 6 months after fingolimod therapy

Parameter	Group	Time point			*p* values			
		Before fingolimod (1)	6 h after fingolimod (3)	6 months after fingolimod (4)	1 vs. 2	2 vs. 3	1 vs. 3	RANOVA
RRI-SD [ms]	Without PHRS	39.9 ± 16.7	48.2 ± 21.3	25.3 ± 10.9	0.004*	< 0.001*	< 0.001*	*p* < 0.001*
	With PHRS	38.7 ± 12.7	55.7 ± 10.9	25.4 ± 9.4	0.035*	< 0.001*	0.014*	
	*p* values	0.991	0.301	0.979				
RRI-CV [%]	Without PHRS	5.1 ± 2.0	5.4 ± 2.2	3.2 ± 1.3	0.242	< 0.001*	< 0.001*	*p* < 0.001*
	With PHRS	5.0 ± 1.6	5.4 ± 1.4	3.0 ± 1.0	0.838	0.001*	0.003*	
	*p* values	0.974	0.933	0.690				
RMSSD [ms]	Without PHRS	30.2 ± 16.4	44.5 ± 23.0	18.0 ± 8.0	< 0.001*	< 0.001*	0.002*	*p* < 0.001*
	With PHRS	25.5 ± 11.8	52.0 ± 15.4	20.0 ± 6.9	< 0.001*	< 0.001*	0.225	
	*p* values	0.493	0.354	0.476				

*Statistically significant (as *p* < 0.05)

**Table 6:** Frequency-domain parameters of 23 patients without prolonged heart rate slowing (PHRS) and 11 patients with PHRS upon fingolimod initiation at three time points: before fingolimod, 6 h and 6 months after fingolimod therapy

Parameters	Group	Time point			*p* values			
		Before fingolimod (1)	6 h after fingolimod (2)	6 months after fingolimod (3)	1 vs. 2	2 vs. 3	1 vs. 3	RANOVA
RRI-LF-powers [ms^2^]	Without PHRS	1147.2 ± 941.5	1402.8 ± 1380.1	556.3 ± 467.6	0.201	0.002*	< 0.001*	*p* < 0.001*
	With PHRS	1076.0 ± 711.7	1987.1 ± 1161.3	495.0 ± 372.1	0.139	0.007*	0.050*	
	*p* values	0.717	0.081	0.971				
RRI-HF-powers [ms^2^]	Without PHRS	428.5 ± 430.8	1031.6 ± 1268.2	121.7 ± 106.8	0.001*	< 0.001*	0.001*	*p* < 0.001*
	With PHRS	326.9 ± 350.6	937.7 ± 491.3	154.4 ± 148.1	0.005*	0.005*	0.131	
	*p* values	0.513	0.451	0.383				
RRI-total-powers [ms^2^]	Without PHRS	1575.7 ± 1300.4	2434.4 ± 2569.1	678.0 ± 525.8	0.006*	< 0.001*	< 0.001*	*p* < 0.001*
	With PHRS	1402.9 ± 906.0	2724.8 ± 1176.4	649.3 ± 498.1	0.047*	0.005*	0.041*	
	*p* values	0.717	0.042*	0.913				
RRI-LF/HF ratio	Without PHRS	4.8 ± 3.9	2.9 ± 4.7	7.6 ± 8.8	0.004*	0.002*	0.346	*p* = 0.057
	With PHRS	9.3 ± 14.1	3.2 ± 2.7	4.5 ± 2.9	0.047*	0.059	0.286	
	Post hoc analysis not performed (ANOVA: *p* > 0.05)							

*Statistically significant (as *p* < 0.05)

**Table 7:** Frequency-domain parameters of blood pressure and baroreflex sensitivity of 23 patients without prolonged heart rate slowing (PHRS) and 11 patients with PHRS upon fingolimod initiation at three time points: before fingolimod, 6 h and 6 months after fingolimod therapy

Parameters	Group	Time point			*p* values			
		Before fingolimod (1)	6 h after fingolimod (2)	6 months after fingolimod (3)	1 vs. 2	2 vs. 3	1 vs. 3	RANOVA
BPsys-LF-powers [mmHg^2^]	Without PHRS	16.4 ± 8.7	14.9 ± 7.6	20.6 ± 13.4	0.378	0.083	0.171	*p* = 0.517
	With PHRS	19.9 ± 12.2	17.5 ± 18.1	17.1 ± 14.6	0.721	0.386	0.722	
	Post hoc analysis not performed (ANOVA: *p* > 0.05)							
BPsys-HF-powers [mmHg^2^]	Without PHRS	3.5 ± 2.0	3.4 ± 3.2	2.8 ± 1.9	0.503	0.761	0.201	*p* = 0.231
	With PHRS	4.4 ± 3.1	2.5 ± 1.9	4.5 ± 4.0	0.074	0.114	0.131	
	Post hoc analysis not performed (ANOVA: *p* > 0.05)							
BRS [ms/mmHg]	Without PHRS	7.4 ± 2.4	10.4 ± 4.5	4.7 ± 2.0	0.002*	< 0.001*	0.001*	*p* < 0.001*
	With PHRS	6.9 ± 3.5	13.1 ± 4.4	5.3 ± 1.4	0.005*	0.005*	0.328	
	*p* values	0.490	0.155	0.214				

*Statistically significant (as *p* < 0.05)

### After 6 months, the 11 patients with initially delayed HR reacceleration had slightly increased parasympathetic/vagomimetic effects

**Fig. 1:**
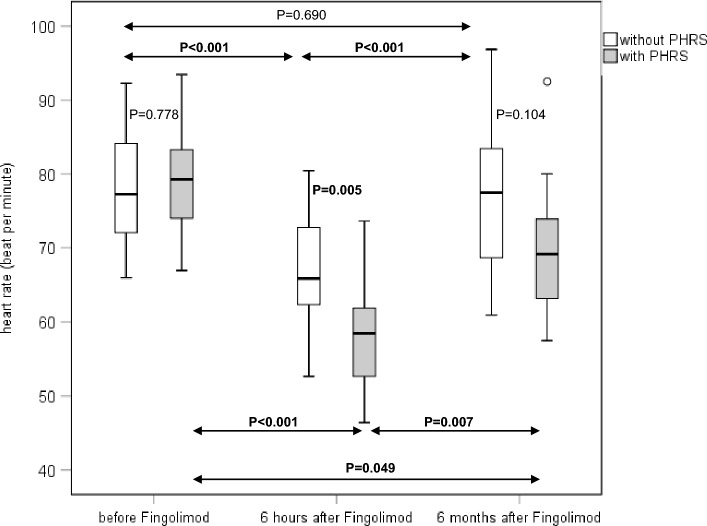
Heart rate of the 23 patients without prolonged heart rate slowing (PHRS) and the 11 patients with PHRS upon fingolimod initiation at three time points: before fingolimod initiation, 6 h after fingolimod initiation, and after 6 months of continuous fingolimod therapy. Data are presented as box plots. The line in the middle of the box represents the median (50th percentile), the top of the box represents the upper quartile (75th percentile), the bottom of the box represents the lower quartile (25th percentile), the end of the whiskers represents the highest and lowest values that are not extreme values or outliers

**Fig. 2:**
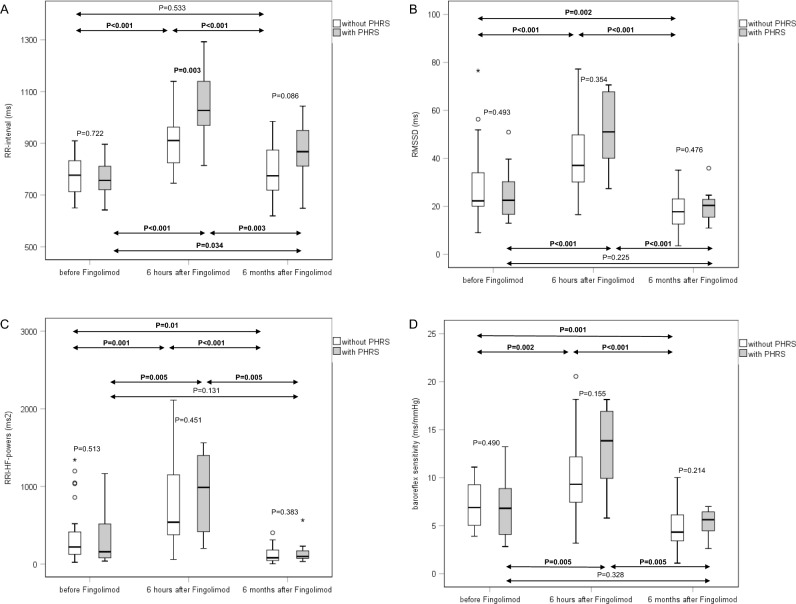
RR intervals, RMSSD, RRI-HF-powers, and baroreflex sensitivity of the 23 patients without prolonged heart rate slowing (PHRS) and the 11 patients with PHRS upon fingolimod initiation at three time points: before fingolimod initiation, 6 h after fingolimod initiation, and after 6 months of continuous fingolimod therapy. Data are presented as box plots as described in Fig. 1

After 6 months of fingolimod therapy, none of the bio-signals or autonomic parameters differed significantly between the patients with and those without prolonged HR slowing (Tables 4, 5, 6, and 7). Moreover, respiratory frequency, BPsys, BPsys-LF-powers, BPsys-HF-powers, and RRI-LF/HF ratios did not differ significantly between the three time points of recording (Tables 4, 5, 6, and 7). In contrast, all other parameters revealed an inversely V-shaped or a V-shaped change from pretreatment values recorded before fingolimod initiation to increased or decreased values 6 h after fingolimod initiation and back towards pretreatment values after 6 months of fingolimod-therapy (Figs. 1 and 2).

In both patient groups, HR values had significantly re-increased and RRIs had decreased compared to 6-h values after fingolimod initiation. However, the 11 patients with prolonged HR slowing still had respectively significantly lower HR values and higher RRI values after 6 months than before fingolimod initiation (Tables 4 and 5) while the 6-month HR and RRI values of the 23 patients without prolonged HR slowing no longer differed from pretreatment values.

In both groups, the 6-month values of RRI-SD, RRI-CV, RMSSD, RRI-LF-powers, RRI-HF-powers, RRI-total-powers, and BRS were significantly lower than the corresponding 6-h values (Tables 4, 5, 6, and 7).

For the parameters of total autonomic modulation RRI-SD, RRI-CV, RRI-total-powers, and for RRI-LF-powers, 6-month values of both patient groups were even significantly lower than the corresponding pretreatment values (Tables 4, 5, 6, and 7). However, the 6-month values of parasympathetic parameters RMSSD, RRI-HF-powers, and of baroreflex sensitivity were significantly lower than the corresponding pretreatment values in the 23 patients without prolonged HR slowing but did not differ from pretreatment values in the 11 patients with prolonged HR slowing upon fingolimod initiation (Tables 4, 5, 6, and 7).

## Discussion

Our results support our hypothesis that not only prolonged HR slowing upon fingolimod initiation but also persistence of HR slowing after 6 months of fingolimod therapy [37–40] may be due to pre-existing changes in central autonomic modulation [42].

### More prominent and prolonged HR slowing upon fingolimod initiation in 11 patients with pre-existing subclinical autonomic changes

The 11 patients who had more prominent HR slowing and delayed HR reacceleration upon the first dose of fingolimod had pre-existing changes in autonomic modulation which were unveiled during orthostatic challenge prior to fingolimod treatment (Tables 3 and 4). The slight autonomic differences cannot be ascribed to differences in age, gender distribution, or disease severity since these parameters were similar between the patients with and those without delayed HR reacceleration (Table 1). In a previous study of 21 patients with RRMS, we identified seven patients who had delayed HR reacceleration upon fingolimod initiation and pre-existing, clinically not overt autonomic dysfunction that became apparent upon autonomic challenge [42]. Similar to these seven patients, our 11 patients with prolonged HR slowing but not the 23 patients who timely reaccelerated HR after fingolimod initiation were unable to significantly withdraw parasympathetic cardiac modulation upon standing up, i.e., upon baroreflex unloading (Table 2 and 3). In contrast to the 23 patients with timely HR reacceleration, parasympathetically mediated RMSSD and RRI-HF-powers did not decrease significantly in the 11 patients upon orthostasis (Tables 2 and 3), and they did not shift autonomic balance from a predominantly parasympathetic cardiac modulation in supine position towards more sympathetic modulation upon standing, i.e., their RRI-LF/HF ratios did not increase significantly upon standing (Table 3) as did the RRI-LF/HF ratios of the 23 patients with timely HR reacceleration (Table 4). In contrast to the 23 patients, the 11 patients with delayed HR reacceleration also had no significant increase in BPsys and BPdia upon standing up (Tables 3). These findings suggest that a slight predominance of parasympathetic cardiac modulation in the 11 patients contributed to their more prominent HR slowing and delayed HR reacceleration upon the first dose of fingolimod. However, parasympathetic RRI modulation was similar in the 11 and the 23 patients not only at baseline, i.e., under resting condition, but moreover did not differ between both groups upon activation of parasympathetic outflow induced by metronomic deep breathing and by BP overshoot upon strain release at the end of VM. Only the ability to lower parasympathetic impact on heart rate upon standing up was compromised in the 11 patients who then showed delayed heart rate recovery after the initial dose of fingolimod. These findings support the conclusion of a subtle parasympathetic predominance with impaired ability to lower the cardiovagal activity adequately upon demand such as baroreflex unloading, or in the presence of a vagomimetic drug such as fingolimod in its early phase as S1P receptor agonist [3–11, 15, 16]. As mentioned above, Akbulak et al. also speculated from their findings of slower H R in patients with RRMS after more than 1 year of fingolimod treatment than before treatment that there might be a pre-existing parasympathetic dominance in patients with RRMS who are at risk of bradycardia and bradyarrhythmia [38].

### Additive effects of centrally mediated parasympathetic and fingolimod-induced HR slowing

The assumption that an inclination towards increased parasympathetic modulation accounts for more prominent and prolonged HR slowing in our 11 patients upon fingolimod initiation or for continued HR slowing after many months of fingolimod treatment, as assumed by Akbulak et al. [38], implies that there are additive effects of fingolimod-induced HR slowing and of centrally mediated parasympathetic HR slowing.

Additive effects have already been postulated by Simula et al. [37]. After 3 months of fingolimod treatment, their 24 patients with RRMS had daytime HRs similar to pretreatment values but nighttime HRs were still lower than before fingolimod treatment, a finding which the authors ascribed to the predominantly parasympathetic HR modulation during sleep [37]. The authors concluded that fingolimod still yields vagomimetic cardiac activity despite several months of medication but inferred from the HR slowing only at night that vagomimetic effects only become apparent with a more prominent parasympathetic modulation, as present during nighttime [37]. This conclusion of additive parasympathetic effects and fingolimod-induced vagomimetic effects on HR is to some extent also supported by the aforementioned findings of Rossi et al. that showed correlations between autonomic parameters assessed before fingolimod initiation and fingolimod-induced bradycardia or PR interval prolongation [41]. DiMarco and co-workers also assumed [36] that timely HR reacceleration not only results from the shift of fingolimod’s agonistic function on cardiac S1P1 receptors towards its antagonistic function due to desensitization and internalization of S1P1 receptors, starting 4–5 h after fingolimod initiation. The authors also postulated that HR counter-regulation additionally depends on other factors including adjustment of—centrally mediated—sympathetic and parasympathetic modulation in response to the initially vagomimetic fingolimod effects [36].

In vitro studies of transmembrane potassium currents in cultured guinea pig myocytes by Bünemann et al. support the concept of additive effects on HR slowing via parasympathetic, acetylcholine-mediated M2-G-protein-coupled receptor activation and via S1P1 receptor activation by endogenous S1P—or by still agonistic fingolimod [10, 17, 27, 31]. The authors showed that simultaneous activation of muscarinic G-protein-coupled M2 receptors by acetylcholine and activation of G-protein-coupled S1P1 receptors by endogenous S1P has additive effects on the current of potassium ions [27, 59].

### Differences in MS-related central lesions seem to account for the subclinical autonomic differences after 6 months

Our finding of a pre-existing slight parasympathetic predominance in 11 patients supports the concept of additive central parasympathetic effects and initially vagomimetic fingolimod effects [60, 61], causing the more prominent HR slowing and delayed reacceleration in the 11 patients 6 h after fingolimod initiation. The 11 patients’ pre-existing propensity towards slightly predominant parasympathetic modulation also explains why they still had lower HRs (and higher RRIs) 6 months after than before fingolimod initiation (Tables 2 and 3), and why parasympathetic RMSSD values, RRI-HF-powers, and baroreflex sensitivity after 6 months had decreased from 6-h values merely to pretreatment values while corresponding values of the 23 patients with timely HR reacceleration had not only decreased from elevated 6-h values to pretreatment values but were even lower after 6 months than before fingolimod initiation, indicating attenuated parasympathetic modulation and baroreflex sensitivity after prolonged fingolimod treatment in these 23 patients (Tables 4, 5, 6, and 7).

According to Chapleau et al., adjustment of baroreflex-mediated sympathetic and parasympathetic output is largely due to changes in central command [61], i.e., the adjustment depends on the ability of the CAN to modify autonomic responses [62]. We suppose that critically located MS lesions [44] interfered with the ability of the 11 patients’ CAN to adequately downregulate parasympathetic outflow in response to baroreflex unloading [60, 61] or to a possible slight persistence of vagomimetic fingolimod effects even after long-term fingolimod treatment [27, 36–38, 41]. Since the autonomic differences between the 11 and the 23 patients were minor and subclinical, they only became apparent upon orthostatic challenge before fingolimod treatment and upon fingolimod initiation.

### Limitations

Our study has some limitations. First, it remains unclear whether fingolimod might have some direct effects on the central autonomic modulation since it crosses the blood brain barrier [1–3, 12]. Such—theoretically possible—central fingolimod effects cannot be dissected from the well-documented and physiologically analyzed direct fingolimod effects at the level of the heart and peripheral vasculature [3–11]. Moreover, we cannot completely rule out that the 11 patients with RRMS might have been more susceptible to the vagomimetic effects of fingolimod. However, at least two observations in our 11 and 23 patients with MS speak against the theory that an increased susceptibility in the 11 patients towards vagomimetic fingolimod effects sufficiently explains the differences between the 11 and the 23 patients with MS of our study. First, it seems rather unlikely that such increased susceptibility occurs in one out of three healthy persons. According to a phase 3b study assessing the cardiac safety during fingolimod treatment, bradycardia adverse events occurred in 0.6% of 2282 patients with MS upon fingolimod initiation [4]. Other studies reported bradycardia in 0.5–2.4%, and atrioventricular blocks in 0.4% of patients with MS upon fingolimod initiation [1, 10]. In contrast to these data, in our study delayed heart rate recovery was recorded in 11 of 34 (32.35%) patients with MS. Second, all of these 11 patients but none of the other 23 patients showed an abnormal autonomic regulation prior to fingolimod treatment which cannot be explained by an interindividual variability of autonomic responses. The 11 patients were unable to significantly lower parasympathetic activity upon standing up, i.e., upon baroreflex unloading. Finally, our patient group was not large enough to attempt to assess associations between the findings of inadequate parasympathetic withdrawal upon baroreflex unloading and the sites of specific MS-related lesions in neuroimages. In a previous study, we were able to associate specific MS lesion sites with increases in sympathetic cardiovascular modulation by means of voxel-based lesion symptom mapping (VLSM) [44]. However, on the basis of our experience from previous neuroimaging studies [44, 63–68], the current study is not adequately powered to apply VLSM and compare parasympathetic responses to baroreflex unloading with the neuroimaging findings of the 11 patients whose heart rate reacceleration was delayed upon fingolimod initiation.

## Conclusion

Several previous studies showed that HR may be slowed in patients with MS even after months or more than 1 year of fingolimod treatment [37–40]. Some of these studies assumed that central autonomic modulation might contribute to the persistence of HR slowing [37–39] despite the fingolimod-induced desensitization, endocytosis, and degradation of the S1P1 receptor–ligand complex [10, 15, 26–28]. Yet, the current study seems to be the first to show that patients with RRMS and enduring HR slowing during long-term fingolimod treatment have a pre-existing, autonomic dysfunction that was not present in the patients with RRMS with timely HR recovery after fingolimod initiation (Tables 2 and 3). Subtle, subclinical parasympathetic predominance, unveiled upon orthostasis-induced baroreceptor unloading, seems to account for the more prominent HR slowing and delayed HR reacceleration upon fingolimod initiation as well as the persistent HR slowing after 6 months of fingolimod treatment (Tables 2 and 3).

There is an additive effect of endogenous S1P—or initially agonistic fingolimod—and of parasympathetically released acetylcholine on G-protein-gated inwardly rectifying potassium channels and the resulting potassium efflux from cardiomyocytes that reduces cardiomyocyte excitability [17, 27, 33, 34]. While this interaction results in the known HR slowing during the first hours after fingolimod initiation due to its agonistic vagomimetic effects, long-term fingolimod intake downregulates and reduces S1P1 receptors [10, 15] and therefore no longer slowed HR after 6 months in our 23 patients who had no pre-existing orthostatic dysregulation. Instead, there was even a central downregulation of parasympathetically mediated RMSSDs, RRI-HF-powers, and of BRS to smaller values after 6 months than before fingolimod treatment (Tables 4, 5, 6, and 7 ). In contrast, the slight parasympathetic predominance, shown in the 11 patients with delayed HR reacceleration and persistent slight HR slowing even after 6 months of fingolimod treatment, prevented a reduction of RMSSDs, RRI-HF-powers, and BRS after 6 months beyond pretreatment values (Tables 4, 5, 6, and 7).

The effect of a more prominent centrally mediated parasympathetic input on HR modulation in the 11 than the 23 patients was, however, minor. After 6 months, it had never resulted in any clinically manifest autonomic dysfunction or cardiac complications. Still, autonomic testing of patients with MS is helpful to identify patients with central autonomic dysregulation prior to disease-modifying therapy and to thereby identify patients with a possibly increased cardiovascular risk upon treatment with an S1P receptor modulator.

While our finding of a selective deficiency in parasympathetic withdrawal upon baroreflex unloading without compromised blood pressure adjustment upon standing up may suggest it to be of minor clinical relevance, the result is of major significance for patients with MS who are about to receive oral treatment with an S1P receptor modulator such as fingolimod. Several studies found associations between pre-existing changes in centrally mediated cardiovascular autonomic modulation [37–39] and prolonged or more pronounced heart rate slowing upon fingolimod treatment [37–40].

Given the high and increasing prevalence of MS, afflicting almost one million patients in the USA [69], and the fact that fingolimod initiation is associated with serious cardiovascular adverse events in 0.9% [1, 3]—theoretically around 9000 patients with MS in the USA—markers that may help identify patients with MS at increased risk of bradyarrhythmias prior to fingolimod treatment are of clinical significance.

Therefore, further studies including for example the evaluation of cerebral blood flow before and during fingolimod treatment might help identify additional changes that could serve as markers of increased risk of bradyarrhythmias upon fingolimod initiation.

## Data Availability

The data that support the findings of this study are available in anonymized dataset from the corresponding author, upon reasonable request.
